# Evaluation of the Presence of Endocrine-Disrupting Compounds in Dissolved and Solid Wastewater Treatment Plant Samples of Gran Canaria Island (Spain)

**DOI:** 10.1155/2013/790570

**Published:** 2013-09-19

**Authors:** T. Vega-Morales, Z. Sosa-Ferrera, J. J. Santana-Rodríguez

**Affiliations:** Departamento de Química, Universidad de Las Palmas de Gran Canaria, 35017 Las Palmas de Gran Canaria, Spain

## Abstract

Liquid and solid samples from two wastewater treatment plants (WWTPs) on Gran Canaria Island (Spain) have been tested for the presence of compounds with endocrine-disrupting properties. The selected degradation stages were sampled bimonthly from each WWTP over the 12-month period from July 2010 to July 2011. The analytical methods used for the determination of the endocrine-disrupting compounds (EDCs) were based on on-line solid phase extraction, microwave-assisted extraction (MAE), and ultrasonic-assisted extraction (UAE) coupled to UHPLC-MS/MS. All of the hyphenated methodologies employed in this work showed good recoveries (72–104%) and sensitivities, with LODs lower than 7.0 ng L^−1^ and 6.3 ng g^−1^ for the dissolved and solid fractions, respectively. We have also evaluated the estrogenicity of the samples in terms of their estradiol equivalent concentrations (EEQs). The chemical analysis of the selected EDCs revealed fairly low concentrations for both natural and synthetic oestrogens, alkylphenolic compounds, and bisphenol-A in each of the dissolved, particulate, and sludge samples (ng L^−1^ or ng g^−1^). However, the estimated estrogenic activity indicated that the majority of samples could represent an important environmental risk, clearly surpassing the threshold to exert deleterious consequences on living beings.

## 1. Introduction

The current concern about endocrine-disrupting compounds (EDCs) is based on scientific facts that collectively indicate that EDCs potential could induce unhealthy changes in humans and wildlife species even at low, ng L^−1^, concentration levels [1–3]. Among EDCs, those that mimic endogenous oestrogens are particularly important because of their central role in reproductive functions [4]. 

 This type of endocrine disrupter, referred herein as estrogenic endocrine disrupting compounds (e-EDCs), interacts with the human oestrogen receptor (hER*α*), which has larger and more flexible binding sites than what natural oestrogen (17*β*-estradiol) requires and is therefore a more vulnerable target for a broad range of interferents with high structural diversity [4]. Moreover, because estrogenic receptors are quite similar between different vertebrates, e-EDCs may affect the endocrine functions of many animal species within the ecosystem. The issue of whether humans and wildlife suffer deleterious consequences resulting from the exposure to these chemicals has been extensively discussed in many reviews (e.g., [5, 6]). 

 There is evidence that prolonged exposure to these substances, even at trace concentrations, could be a causal factor in breast cancer [6] and testicular germ cell cancer [7]. e-EDCs have also been correlated to increased rates of hypospadias and cryptorchidism [8] as well as the decreasing sperm count observed in many countries [9]. 

 The effects of these substances on wildlife species have also been shown in numerous scientific papers [10–12] with similar expectations. In short, several alterations have been reported in a wide range of animal species, such as decreased fertility in birds, fish, and mammals; demasculinisation and feminisation of fish, shellfish, gastropods, birds, and mammals; reduced efficacy of the incubation processes in fish, birds, and turtles; immune system disorders in birds and mammals; and alterations in the thyroid of birds and fish [13]. 

 e-EDCs can enter the environment from a variety of sources. However, the vast majority of them are generally focused in localised “hot spots” of chemical discharges, such as wastewater treatment plants (WWTPs) effluents, agricultural runoff, and landfills, which create a continuous input of these pollutants into the environment [14]. Despite this fact, the relatively high lipophilicity and persistence of e-EDCs enable their bioaccumulation and biomagnification within the environment, and therefore, a more globalised phenomenon of endocrine disruption is possible.

 The primary objective of the present work was to develop a comprehensive evaluation of the endocrine-disrupting activity in different fractions of wastewater samples (dissolved phase, particulate phase, and sludge) taken from two different wastewater treatment plants (WWTPs) on the island of Gran Canaria (Spain). The chemicals analysed in this work included both natural and synthetic steroids as well as nonsteroidal compounds (Table 1). Regardless of their nature, all of the selected compounds share the ability to mimic endogenous estradiol and have been frequently found in both WWTP effluents and surface waters. To achieve our objective, we evaluated the estrogenicity of the samples in terms of their estradiol equivalent concentrations (EEQs) [15, 16]. Because the effects of estrogenic activity in e-EDCs have proved to be additive, the EEQ can be defined as the sum of the concentrations for each individual e-EDC after normalising by an estradiol equivalency factor (EEF) [17]. These EEFs are defined as the quotient of half maximal effective concentrations of estradiol and other EDC (EC50_E2_/EC50_EDC_) and are set to 1 for estradiol (E2) [18].

To estimate the concentration of each e-EDC, we employed a variety of extraction/purification procedures that have been previously published by our research group [19, 28, 29]. These methods include microwave-assisted extraction (MAE) for sludge samples, ultrasonic-assisted extraction (UAE) for particulate matter, and on-line solid Phase Extraction (SPE) for the dissolved fraction. All of these extraction techniques were subsequently coupled to an UHPLC-MS/MS instrument for the identification and quantification of each compound. 

In turn, the calculations of the EEFs were carried out by averaging the most recent values reported in the scientific literature. Given the wide range of EEF values observed even amongst the same e-EDC, we considered several biological, based assays (BBAs), such as the oestrogen receptor (ER) binding assay, the yeast oestrogen screen (YES), and the oestrogen responsive chemically activated luciferase expression (ER-CALUX), to obtain significant EEFs for each analyte being studied. This approach has been successfully employed before in other publications [17, 30].

 To the best of our knowledge, this is the first attempt to estimate the potential estrogenic risks in this region and one of the few reports [17, 21, 31] that take into consideration the different fractions of WWTPs samples that are released into the environment.

## 2. Method and Materials

### 2.1. Chemicals

 The natural oestrogens 17*β*-estradiol (E2), estrone (E1), and Estriol (E3); synthetic steroids 17*α*-ethinylestradiol (EE) and diethylstilbestrol (DES); and nonsteroidal compounds bisphenol-A (BPA), nonylphenol (NP), and octylphenol (OP) were purchased from Sigma Aldrich in greater than 98% purity (Madrid, Spain). Stock solutions were prepared at 1000 *μ*g mL^−1^ by dissolving each standard in methanol and storing in glass-stoppered bottles at −18°C. 

 Short ethoxylated chains (AP_1-2_EOs) were directly acquired as stock solutions (10 *μ*g mL^−1^ in 1 mL) in acetone and stored at −18°C. Long-chained AP_*n*_EOs (*n* ≥ 3) were only available in technical mixtures. Igepal CO210, CO520, and CO720 contained a range of NP_*n*_EO oligomers with 3–12 ethoxy units (EO), whereas Igepal CA210, CA520 and CA720 contained the same EO range of OP_*n*_EO oligomers. Stock solutions (1000 *μ*g mL^−1^) of long-chain alkylphenolic ethoxylated surfactants were also prepared by dissolving the appropriate quantities. These solutions were used to calculate the concentrations of the analysed samples; however, considering their low to nonexistent estrogenic potential, we have not included them in the EEQ calculations.

### 2.2. Sampling Sites and Collection

 Samples of wastewater, particulate matter, and sludge were collected bimonthly for a period of 12 months (July 2010–July 2011) from two different WWTPs located in northeastern Gran Canaria island (Spain). This region contains both the vast majority of the population (hosting more than half a million people) and the largest concentration of the limited industrial activity on the island. Therefore, domestic sewage systems are the primary source of raw wastewater flowing into the two WWTPs. The characteristics of each WWTP under study are described below.

*WWTP1*. WWTP1 is based on a conventional activated sludge (CAS) process. In this plant, there was an important agricultural water input (irrigation of agricultural crops) flowing into the plant despite the predominantly domestic nature of the incoming wastewater. This plant possesses a treatment capacity of 10 L seg^−1^, or approximately 5,000 equivalent inhabitants. Liquid wastewater samples were taken from the output of the secondary treatment, the clarifier-settled tank effluent, whereas the sludge samples were collected from drying tanks that were exposed to the outdoors.

*WWTP2*. WWTP2 employed a novel biomembrane reactor (BMR) treatment process. WWTP1 and WWTP2 receive very similar raw wastewaters with an important agricultural water input given the similarities between both locations. The treatment capacity of this plant slightly surpassed 7000 equivalent inhabitants. In this case, the liquid wastewater samples were taken after the biomembrane filtration process, whereas the sludge samples were collected from the output of the centrifuge used to dewater the sludge. 

### 2.3. Sample Pretreatment

 The liquid wastewater samples were collected in glass stoppered amber bottles (2.5 L) and acidified to a pH of <3 to prevent the loss of the e-EDC targets via biological degradation and abiotic reactions such as hydrolysis [32]. The samples were then stored at 4°C and extracted within 48 h. Prior to this extraction, the wastewater samples were filtered through 0.45 *μ*m membrane filters (Millipore, Bedford, MA, USA). This pore size was used as a threshold to separate the dissolved and particulate phases [33]. 

 Therefore, the 0.45 *μ*m filters with the retained particulate matter (between 0.1 and 0.2 g for all samples) were stored at −18°C prior to analysis. Sludge samples were collected in glass-stoppered flasks and stored in a freezer at −18°C. The target compound determination of both the particulate matter and sludge samples was conducted within 48 hours. 

### 2.4. Analytical Methods

 In the evaluation of the estrogenicity of the environmental samples, the calculation of the e-EDC concentrations was the first goal achieved. We employed three extraction/preconcentration methods that had been previously published by our research group to accomplish this goal [19, 28, 29]. 

All of the methods's hyphenated methodologies employed in this work showed the sensitivity and selectivity needed to determine this class of compounds in wastewater matrices (Table 2). The recoveries for both liquid and solid samples were between 72–104%, whereas the limits of detection ranged from 0.3 to 2.1 ng L^−1^ in liquid samples, and from 0.1 to 1.9 ng g^−1^ in solid samples, including both particulate and sludge materials. In addition, the triple quadrupole mass spectrometry detection system employed in this work offered the selectivity criteria (e.g., retention times, parent and product ions, and ion ratios) needed to unequivocally determine the selected compounds. 

 In order to take into consideration the matrix effects, a well-known phenomenon which usually impair the proper ionization of the analytes in complex matrices when using ESI interfaces, we have employed in each methodology a matrix-matched calibration for the quantification of the analytes. This approach allowed us, by the one hand, to not underestimate the results due to the ion suppression observed in the electrospray interface, and on the other, to prevent the use of isotopic labeled internal standards for the quantification processes. This latter case, although it is probably the most appropriate approach, requires an important investment due to the high cost of acquisition, and often are not commercially available, as occured with several of our analytes (e.g., alkylphenolic polyethoxylated compounds).

**Figure 1:**
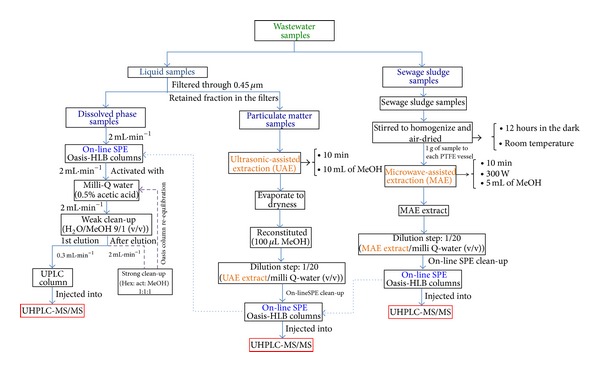
Flow scheme of the analytical methodologies that have been undertaken for the chemical analysis of the selected e-EDCs in each fraction.

A brief description of each method is given in the following sections.  Figure 1 shows the pathways undertaken to analyse the e-EDCs in all of the fractions.

#### 2.4.1. Analysis of e-EDCs in Liquid Samples

The accurate and simultaneous determination of the selected e-EDCs dissolved in the wastewater samples was undertaken first. The analytical method consisted of an on-line SPE step followed by the determination of the selected compounds via ultra-high performance liquid chromatography coupled to a triple quadrupole mass spectrometry detector (UHPLC-MS/MS) (Waters, Milford, MA, USA). This method enabled us to considerably improve the limits of detection and quantification relative to the off-line SPE methods. Moreover, it significantly reduces both the global analysis time and background noise and noticeably improves the reproducibility of the results.

 The on-line SPE protocol was performed using two Oasis HLB extraction columns (20 *μ*m, 2.1 mm × 50 mm) working in parallel. Chromatographic separation was accomplished using an ACQUITY BEH C_18_ chromatographic column (1.7 *μ*m, 2.1 mm × 50 mm). Both the chromatographic and extraction columns were acquired from Waters (Milford, MA, USA). The mobile phase consisted of both water and methanol containing 0.1% NH_3_ to promote both the proper ionisation of the compounds in the electrospray interface (ESI) and the formation of ammonium adducts. The gradient elution consisted of a 50 : 50 (v : v) mixture of water:methanol that was linearly increased to 100% methanol (B) over 4 minutes [19].

#### 2.4.2. Analysis of e-EDCs in Solid Samples

*Sludge Samples*. The concentrations of these substances in the sewage sludge samples were obtained using a microwave-assisted extraction (MAE) technique [28], followed by the On-Line-SPE-UHPLC-ESI-MS/MS protocol described in the previous section [19] to purify the extracts and determine the concentration of each analyte.

 To summarise the MAE procedure, 1 g of the sludge was transferred to a polytetrafluoroethylene (PTFE) vessel. Next, 5 mL of an extractant (methanol) was added to the sample, and the vessels were closed and placed symmetrically on a rotor. Once the rotor was placed in the microwave oven, a power of 300 W was used for 10 min.

*Particulate Matter*. The extraction of the e-EDC from the particulate phase was carried out according to the following extraction methodology [29].

 The 0.45 *μ*m membrane filters with the retained particulate matter (between 0.1 and 0.2 g for all samples) were immersed for 10 min in an ultrasonic bath containing 10 mL of methanol. The methanol extract was collected in a flask, evaporated to dryness under a gentle nitrogen stream, and reconstituted in 100 *μ*L of methanol. The final extracts were analysed separately, and the concentrations of the dissolved and particulate phases are reported separately for each sample. The determination of the analytes was accomplished via On-Line-SPE-UHPLC-ESI-MS/MS [19]. 

### 2.5. Estrogenic Potential Calculations

The estrogenicity of the samples was evaluated in terms of the estradiol equivalent concentrations (EEQs) [15, 16]. EEQs can be defined as follows:

(1) $$\begin{array}{c} {\text{EEQ}}_{i}=C_{i}\times {\text{EEF}}_{i}, \end{array}$$

where *C*_*i*_ is the concentration of compound *i* in the sample, and EEF_i_ is the estradiol equivalency factor of compound *i*. 

#### 2.5.1. Estradiol Equivalency Factors

The estradiol equivalency factor (EEF) is defined by the following expression:

(2) $$\begin{array}{c} {\text{EEF}}_{i}=\frac{{\text{EC}50}_{\text{E}2}}{{\text{EC}50}_{i}}, \end{array}$$

where EC50_E2_ is the concentration that yields half of the maximum response for the estradiol, and EC50_E2_ is the concentration that yields half of the maximum response for compound *i*.

 A large number of in vitro tests have been developed for the rapid and sensitive screening of endocrine disrupting chemicals [34]. However, the most widely employed in vitro tests for estimating the estrogenic potential and thus the estradiol equivalency factors can be summarised as follows.

*(i) Receptor Binding Assays*. Measure the binding affinity of a substance to a hormone receptor (e.g., oestrogen receptor (ER)).

*(ii) Cell Proliferation Assays*. Measure the ability of a substance to stimulate the growth of a hormone responsive cell (e.g., MCF-7, and E-screen).

*(iii) Reporter Gene Assays*. Measure the ability of a substance to activate the transcription of a reporter gene construct in cells (e.g., yeast oestrogen screen (YES) or mammalian cell assays).

All of these in vitro tests are able to satisfactorily estimate the estrogenicity of several e-EDCs in a large variety of environmental matrices such as the wastewater samples. However, the EEFs reported in the literature for the same e-EDC vary significantly depending on the in vitro test employed [20, 23, 35]. In addition, the reported EEFs sometimes vary significantly for the same in vitro test applied by different researchers [14].  Table 3 shows the EEFs previously reported for the selected e-EDCs in the literature. 

 Given the observed variability, the EEFs reported in the literature were averaged, and then, applied in (1) to calculate the individual estradiol equivalent concentrations (EEQ_*i*_).

#### 2.5.2. Estrogenic Potential

As the additive estrogenic activity of e-EDCs has been proven [17], the EEQ can be defined as the sum of the concentrations for each individual e-EDC after normalising with their estradiol equivalency factors (EEFs). Thus, the concentrations obtained from the analytical methods employed (*C*_*i*_) for each target e-EDC and fraction studied (dissolved phase, particulate matter and sludge) were multiplied by their relative potency.

Consider

(3) $$\begin{array}{c} {\text{EEQ}}_{t}=\sum {\text{EEQ}}_{i\;},\\ {\text{EEQ}}_{t}=\sum \left[C_{1}+{\text{EEF}}_{1}\right]+\left[C_{2}+{\text{EEF}}_{2}\right]+\left[C_{3}+{\text{EEF}}_{3}\right]\cdots . \end{array}$$

The estradiol equivalent concentration calculated from the sum of the individual compounds represents the overall endocrine-disrupting activity of the sample.

## 3. Results and Discussions

### 3.1. Concentrations of Selected EDCs

 The concentrations of the recorded e-EDCs for all of the samplings conducted (July 2010–July 2011) are highlighted in Tables 4 and 5. Each table shows the concentration of the selected analytes in the dissolved phase fraction (samples are collected from the final effluents of the two WWTPs under study) and solid phases (particulate matter and sludge samples), respectively.

#### 3.1.1. Natural and Synthetic Oestrogens

The concentration of both natural and synthetic oestrogens dissolved in the wastewater samples has consistently been in the low ng L^−1^ levels throughout the entire sampling time. 

 Of the selected natural oestrogens, 17*β*-estradiol (E2) and estriol (E3) were present in the highest concentrations in the two WWTPs studied with an average of 21.5 and 16.8 ng L^−1^, respectively. Moreover, both compounds were consistently detected in the liquid samples (93% of all cases). Estrone (E1) was also detected at low ng L^−1^ levels in these fractions; however, neither its average concentration (5.4 ng L^−1^) nor its detection frequency (detected in only 50% of the samples) approached those reported for E2 and E3. 

 The studied synthetic steroids, 17*α*-ethinylestradiol (EE) and diethylstilbestrol (DES) were only detected in a few samples of this fraction (14% and 7% of the total samples, resp.). However, they were regularly found in both the particulate matter and sludge samples with observed concentrations higher than those of natural oestrogens. This phenomenon led us to hypothesise that, given the relatively high octanol/water partition coefficient and low water solubility of EE and DES, both substances were strongly associated with the solid fractions of the samples; however, they were not eliminated by the WWTP degradation processes.

 The natural and synthetic oestrogen concentrations observed in both the particulate matter and sludge samples were in the low ng g^−1^ levels, reaching concentrations close to 100 ng g^−1^ in a few cases. Attending to the obtained results, we can describe a common behavioural pattern for these substances; compounds with a relatively high log *K*_ow_ were systematically detected in the solid fraction of the samples (e.g., EE, DES or E2), whereas compounds with a relatively low log *K*_ow_ were not commonly detected in either the particulate matter or sludge samples (e.g., E3).

 The analytical results obtained in the present study for oestrogens in water are in agreement with those of previous studies [17, 18, 36].

#### 3.1.2. Alkylphenolic Compounds and Their Ethoxylates

Alkylphenolic ethoxylated surfactants (AP_*n*_EOs) and raw alkylphenols (APs) (nonylphenol (NP) and octylphenol (OP)) were commonly found in all of the studied matrices in concentrations ranging from low parts per trillion (ppt) to low parts per billion (ppb).

 The distribution of the alkylphenolic ethoxylates varied significantly between the different matrices analysed. On the one hand, we detected the presence of a wide range of AP_*n*_EOs in the dissolved samples, ranging from raw nonylphenol and octylphenol to ethoxymers containing 1 to 12 ethoxylated units. On the other hand, we consistently determined the presence of nonylphenol, octylphenol, and short-chained AP_*n*_EOs (*n* < 5) in the solid matrices; however, concentrations of the more water-soluble ethoxymers (*n* > 5) were only found in a small percentage of these samples. 

 While it would be more logical to find higher concentrations of the more water-soluble compounds, such as long-chained AP_*n*_EOs (*n* > 5), in the dissolved phase, that was not the case for any of the WWTPs. Considering that we have been analysing samples of the final effluent, this phenomenon can be explained as a direct consequence of AP_*n*_EO breakdown in the WWTPs, most likely during biological treatments [37]. These compounds progressively lose ethoxylated units, which form APs (a raw material for microorganisms), short-chain AP_*n*_EOs, and other biotransformation products such as carboxylated and halogenated derivatives [38].

 Therefore, as the degradation treatments progressed, the relative composition of the homologous mixture was further enriched with short-chained AP_1-2_EOs and APs, which are more toxic, more lipophilic, more estrogenic, and more persistent than the parent substances [39]. As explained above, these substances tend to be quickly adsorbed by the particulate matter present in the samples. We estimated that over 80% of the total NP and 60% of total OP were found in the particulate phase, which indicates the importance of analysing these compounds in the solid fraction.

#### 3.1.3. Bisphenol-A

 Bisphenol-A (BPA) was consistently found in the dissolved phase (65% of the samples conducted) in concentrations ranging from 4.9 ng L^−1^ to 39.5 ng L^−1^. However, its presence in the solid fractions seems to be much more erratic. We only observed BPA concentrations above the quantification limits in 57% of the sludge samples and 35% of the particulate matter samples analysed. The average BPA concentration in the particulate matter was 11.2 ng g^−1^, whereas its average concentration in the sludge samples was 5.8 ng g^−1^. This behaviour can be attributed to its relatively low octanol/water partition coefficient (see Table 1).

#### 3.1.4. Evaluation of the Temporal Variability

Regarding the temporal evolution recorded over the 12 months of sampling, we can state that in most cases, it was not possible to link the fluctuations in the concentrations of the studied compounds with the changes of the physical parameters associated with seasonal variations, or with the volumes and characteristics of the inlet waters, or even with the different biological treatments employed in both WWTPs, having been observed a great randomness in the results obtained. This high variability can also be attributed to the different origins and uses of the selected compounds (both natural (e.g., E2 and metabolites) and anthropogenic (e.g., BPA, DES or EE)).

### 3.2. Estimation of Estrogenic Activity

The field experiment results showed us that a complex mixture of endocrine-disrupting compounds occurs in the effluent from the studied WWTPs that will eventually enter the environment where aquatic organisms are exposed to these pollutants. Moreover, we detected that the most estrogenic compounds are strongly linked to the particulate matter and, therefore, to the sludges used during the biological treatment. This fraction should also be viewed as a potential emission source of these substances into the environment via landfills or as organic amendments to mitigate the low productivity or profitability of several agriculture soils, a technique that has become a common practice in Europe [40].

 Based on these facts, the next step responds to these questions: how estrogenic are the subproducts generated by the two studied WWTPs?, how estrogenic are the different analysed fractions?, and how could these emissions affect living beings in the impacted areas? The reported concentrations cannot answer these questions by themselves, so we were encouraged to go one step further and estimate the estrogenic potential of the dissolved, particulate matter and sludge samples from each of the WWTPs studied. The estrogenic potential calculations were conducted in terms of estradiol equivalent concentrations (EEQs) as was explained in detail in Section 2.5.

#### 3.2.1. Liquid Samples

The deleterious consequences resulting from exposure to e-EDCs have been reported for in vivo experiments using estradiol concentration as low as 1 ng L^−1^ [22, 41]. Therefore, we selected this threshold (EEQs of 1 ng E2 L^−1^ in liquid samples or 0,001 ng E2 g^−1^ in solid matrices) as the expected effect concentration to catalogue a sample as a potential environmental risk due to its estrogenic potency. The EEQs calculation, in addition to allowing us to determine the estrogenic potency of the selected e-EDC mixture, also has the capacity to estimate the average contribution of each analyte to the estradiol equivalent concentration (EEQ), which enables us to identify the compounds contributing the most to the total estrogenic potential.

**Figure 2:**
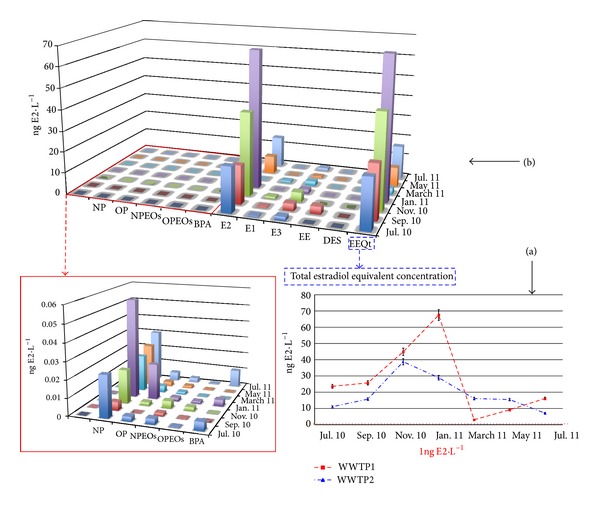
(a) Total EEQs obtained in the dissolved phase fraction for the two WWTPs in each sampling conducted. (b) Contribution of each e-EDC to the estrogenic potential registered for the WWTP 1 (July 2010–July 2011) in the dissolved fraction.

Figure 2(a) showed the contribution of each e-EDC to the estrogenic potential of the dissolved fraction from WWTP1 (July 2010–July 2011). The total EEQs obtained in this fraction for each sampling conducted and in both WWTPs are displayed in Figure 2(b). 

As indicated by the EEQs values reported in Figure 2(a), the estrogenic potential of almost all of the samples significantly surpassed the threshold effect concentration of 1 ng E2 L^−1^. Despite the differences between the equivalent inhabitants treated at each WWTP and the degradation mechanisms employed, we did not observe a significant difference between the EEQs obtained for each one: WWTP1 (3.0–44.9 ng E2 L^−1^) and WWTP2 (7.1–38.7 ng E2 L^−1^). 

Considering the results shown in Figure 2(b), it could be stated that the greatest (and almost exclusive) contributing compound to the total EEQ is natural estradiol (E2), which was detected in almost all of the analysed samples. The rest of the e-EDCs did not exert a large influence on the estrogenic potential despite having higher concentrations than E2 in the majority of the cases. This situation can be explained because of their low EEFs relative to that of E2, which is set to 1. 

 Nonylphenol (NP) and octylphenol (OP) have been catalogued as the most estrogenic alkylphenolic substances and are even included in the list of potentially hazardous compounds [18]. However, it is important to emphasise that OP and NP are not themselves estrogenically active as their potentials are 100 to 1000 times lower than the set limit. BPA showed a similar pattern to that was observed for both alkylphenols: its low EEF prevents significant EEQ values unless large concentrations were presented into the samples. In addition, the concentration of BPA in this type of sample was less important than those reported for NP and OP. The EEQs obtained for both the alkylphenolic compounds and BPA are highlighted in Figure 2(b).

#### 3.2.2. Solid Samples

 Before now, little attention had been paid to the contribution of particulate matter and sludge materials to the total estrogenic potency of wastewater samples, even though more lipophilic compounds could more easily reach biota via the food chain. Ying and Kookana recently studied the degradation of BPA, E2, E1, EE, OP, and NP in soils [42], seawater, and marine sediments [43]. In both papers, they concluded that all of the e-EDCs analysed, including the degradation product E1, degraded under aerobic conditions within 4–20 days (as also occurs in the dissolved samples). However, under the anaerobic conditions that occur in landfills or sediments adjacent to sewage outfall, little or no degradation of the six e-EDCs was found except for E2, which showed slowly degraded during the 70 d study. The calculated half-life for E2 under anaerobic conditions was 24 days in soil. They also found that E2 was biotransformed to E1 under both aerobic and anaerobic conditions, which suggests a progressive reduction of the estrogenic potential as time progressed.

**Figure 3:**
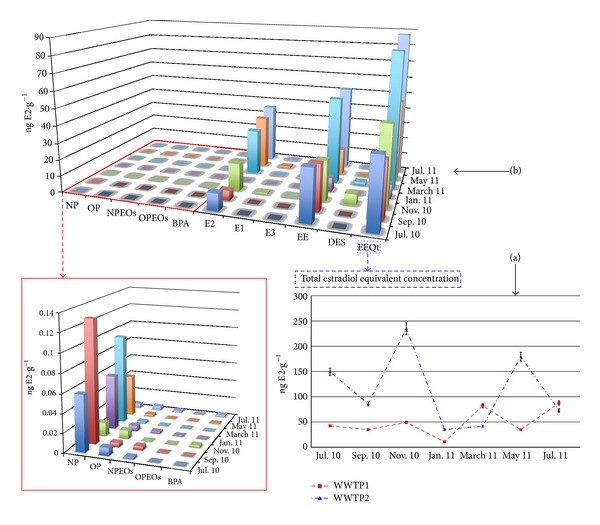
(a) Total EEQs obtained for the sludge samples in the two WWTPs during the whole sampling period. (b) Contribution of each e-EDCs to the estrogenic potential registered for the WWTP 1 (July 2010–July 2011) in the sludge samples.

*Sludge Samples.* The estimated EEQs of the solid samples can be determined in the same way adopted for the liquid samples. In addition, the EEQs of these kinds of matrices have been previously calculated in other publications [14, 36]. Both the obtained estrogenic potencies and the contribution of each analyte (WWTP1) are presented in Figures 3(a) and 3(b), respectively. 

Interestingly, the sludge samples contained large amounts of estrogenic compounds, especially those with higher EEFs. The constant presence of EE and DES significantly increases the estrogenic potency of these samples with respect to the dissolved phase. In addition, EE was the greatest contributing compound to the estrogenic potency for the majority of the samples (Figure 3(b)). 

 Although much higher concentrations of NP, OP, and their short-chained ethoxylated compounds were found in this fraction, their contribution to the estrogenic potency remained almost negligible, just as in the dissolved phase. The average contribution of entire AP_*n*_EOs family to the total EEQ was less than 0.3%. 

BPA showed similar behaviour to the alkylphenolic compounds with an average contribution below 0.1%. EEQ values ranging from 8.6 to 238.8 ng E2 g^−1^ were obtained for this fraction. These potentials largely exceed the EEQs values determined for the dissolved phase from the same sampling. The results obtained from each station were similar, although the potentials observed for WWTP1 were slightly lower (Figure 3(a)).

**Figure 4:**
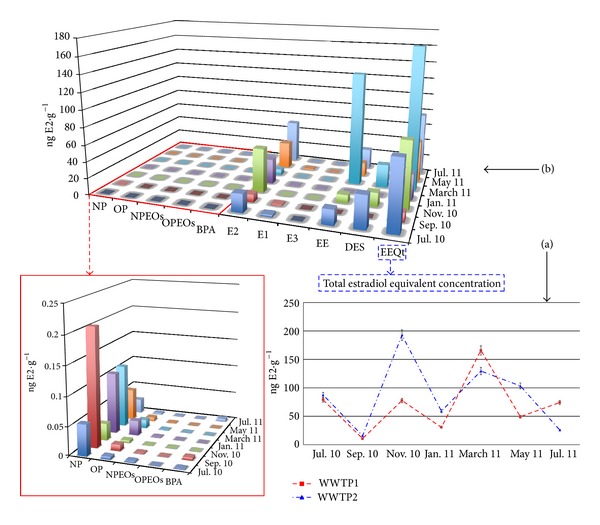
(a) Total EEQs obtained for the particulate matter samples in the two WWTPs during the whole sampling period. (b) Contribution of each e-EDCs to the estrogenic potential registered for the WWTP 1 (July 2010–July 2011) in the particulate matter samples.

*Particulate Matter Samples.* The measured estrogenic activity for particulate matter was between 10.8 and 192.8 ng E2 g^−1^, which was slightly lower than those reported for the sludge samples. The average contribution of each e-EDC to the estrogenic activity was the same with EE as the most contributing compound closely followed by E2. DES also possessed an important contribution to the total estrogenic potency with an average of approximately 20% in each station. During July 2010 sampling, DES was the most contributing compound to the total EEQ (approximately 50%). The behaviour between both WWTPs was similar to that reported for the sludges. The results obtained are displayed in Figures 4(a) and 4(b). 

## 4. Conclusions

 In the present work, the endocrine-disrupting activity of wastewater samples collected from two WWTPs from the island of Gran Canaria (Spain) was evaluated in terms of estradiol equivalent concentrations (EEQs). The chemical analysis of the selected e-EDCs revealed fairly low concentrations for both natural and synthetic oestrogens, alkylphenolic compounds, and bisphenol-A in each of the analysed fractions (ng L^−1^ or ng g^−1^). However, the estimated estrogenic activity indicated that the majority of samples strongly surpassed the threshold concentration, especially the particulate fraction and sludge samples, which indicates a potential environmental risk.

 Because of the lipophilic nature of the compounds with the greatest contributions to the total EEQ values, it would be a mistake to estimate the estrogenic potential of environmental samples exclusively from the dissolved phase, which occurs in most cases. From our results, we can deduct that the solid fraction, particulate matter and sludge, and therefore sediments pose a much higher “*estrogenic charge*” than observed for the liquid samples. Moreover, the solid fraction presents a more direct entry pathway to living beings via the food chain, which also supposes a higher environmental risk.

## Figures and Tables

**Table 1:** Physicochemical properties of the compounds under study.

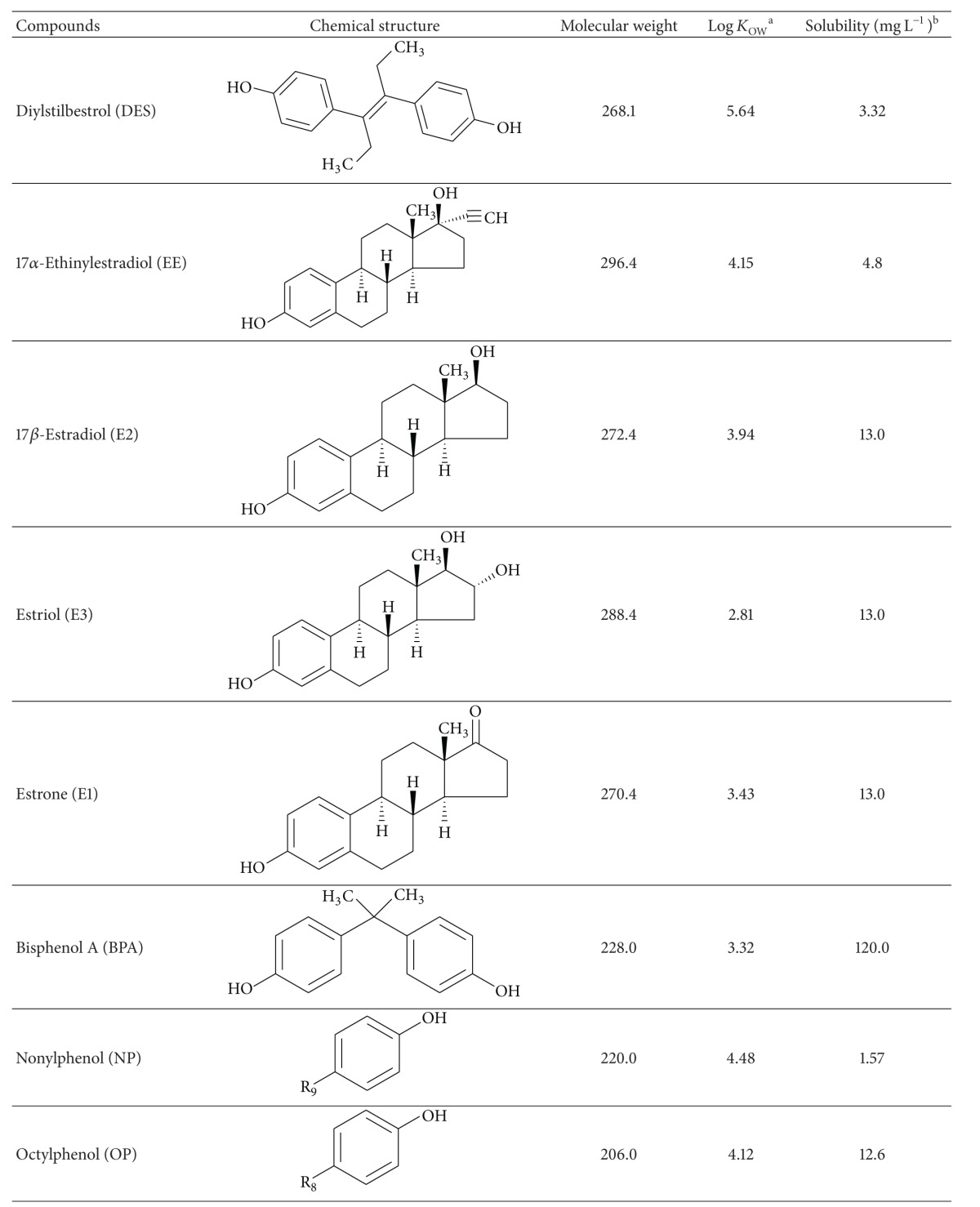 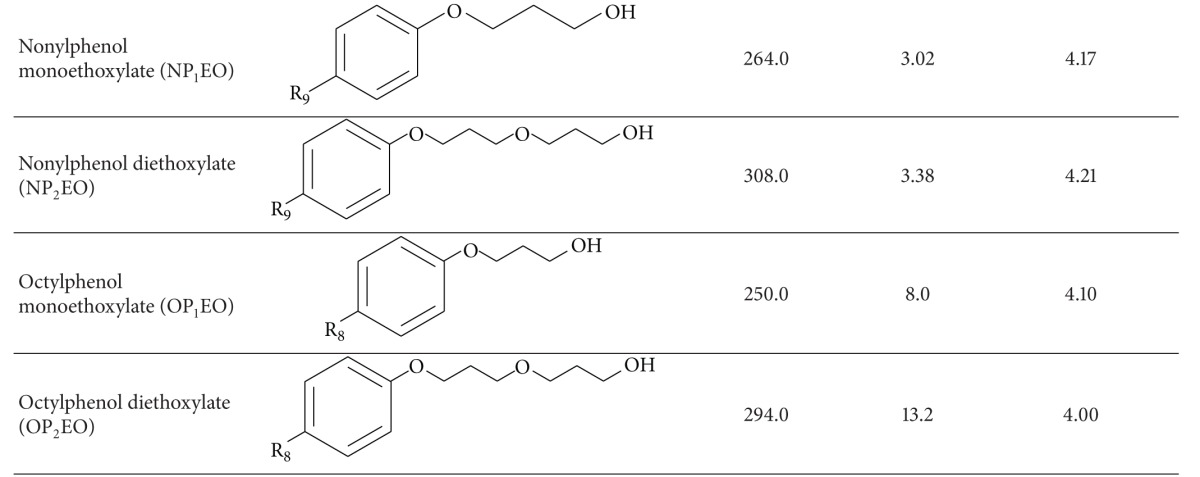

^a^Octanol/water partition coefficients. ^b^Solubility at 20°C.

**Table 2:** Analytical parameters obtained for the developed methodology.

On-Line-SPE-UHPLC-MS/MS^e^						
Compound	LOD^a^ (ng L^−1^)	LOQ^b^ (ng L^−1^)	RSD^c^ (%, peak area)		Recovery^d^ (%)	
			5 ng L^−1^ (*n* = 6)	50 ng L^−1^ (*n* = 6)	10 ng L^−1^ (*n* = 6)	500 ng L^−1^ (*n* = 6)
NP	1.3	4.3	1.7	5.2	93.1	95.3
OP	1.8	6.0	2.4	9.8	89.9	95.0
NPEOs	0.3–1.8	1.0–6.0	1.1–3.9	2.0–8.1	83.1–100.2	84.1–104.0
OPEOs	0.7–2.1	2.3–7.0	0.9–4.8	1.5–7.3	86.2–104.2	85.3–95.7
BPA	1.9	6.3	5.0	8.5	90.7	88.4
E2	1.0	4.0	6.4	5.7	89.7	88.4
E1	1.3	4.3	2.1	4.9	84.5	98.3
E3	1.3	4.3	7.7	10.1	86.9	85.4
EE	0.9	3.0	6.0	9.1	88.3	90.9
DES	0.6	2.0	7.5	6.2	102.5	90.3

MAE-On-Line-SPE-UHPLC-MS/MS						
Compound	LOD^a^ (ng g^−1^)	LOQ^b^ (ng g^−1^)	RSD^c^ (%, peak area)		Recovery^d^ (%)	
			5 ng g^−1^ (*n* = 6)	250 ng g^−1^ (*n* = 6)	5 ng g^−1^ (*n* = 6)	250 ng g^−1^ (*n* = 6)

NP	0.1	0.3	8.9	9.2	79.5	78.2
OP	0.1	0.3	7.0	8.1	82.6	88.1
NPEOs	0.1–0.4	0.3–1.3	1.3–7.1	2.1–8.7	74.3–99.7	76.7–102.0
OPEOs	0.1–0.6	0.3–2	0.9–5.7	1.9–6.3	82.5–100.7	80.3–95.1
BPA	0.2	0.7	3.3	3.3	79.3	82.2
E2	0.3	1.0	5.0	8.1	95.3	90.4
E1	0.5	1.7	4.8	5.7	75.1	80.9
E3	0.1	0.2	2.3	4.0	88.8	83.9
EE	0.3	1.0	8.3	5.9	98.1	97.2
DES	0.1	0.3	5.8	5.4	92.9	97.0

UAE-On-Line-SPE-UHPLC-MS/MS						
Compound	LOD^a^ (ng g^−1^)	LOQ^b^ (ng g^−1^)	RSD^c^ (%, peak area)		Recovery^d^ (%)	
			25 ng g^−1^ (*n* = 6)	250 ng g^−1^ (*n* = 6)	25 ng g^−1^ (*n* = 6)	250 ng g^−1^ (*n* = 6)

NP	1.3	4.3	3.7	3.1	71.5	73.2
OP	0.9	3.0	6.1	8.4	77.7	80.9
NPEOs	0.3–1.8	1.0–6.0	0.9–5.0	0.7–4.2	81.2–102.6	77.3–96.5
OPEOs	0.6–1.9	2.0–6.3	1.2–6.1	0.5–4.2	79.9–103.5	80.9–98.4
BPA	0.5	1.3	3.3	6.1	85.2	90.0
E2	1.3	4.3	5.0	4.6	94.3	96.4
E1	1.0	4.0	4.8	8.0	85.1	92.4
E3	0.9	3.0	2.3	7.3	100.2	98.6
EE	1.9	6.3	8.3	4.9	91.4	87.3
DES	0.5	1.3	5.8	3.5	86.1	90.2

^a^Limit of detection; ^b^limit of quantification; ^c^repeatability (intraday assays) expressed as relative standard deviation (RSD); ^d^recovery percentages obtained for different matrices spiked at two different concentration levels; ^e^data already published in Vega-Morales et al., 2012 [19].

**Table 3:** Estradiol equivalency factors values of 17*β*-estradiol, estrone, estriol, 17*α*-ethinylestradiol, diethylstilbestrol, bisphenol-A, nonylphenol, octylphenol, and their short-chained ethoxylates.

	E2	E1	E3	EE	DES	BPA	NP	NP_1_EO	NP_2_EO	OP	OP_1_EO	OP_2_EO
EEF^a^	1^b^	0.38^b^	2.4 × 10^−3^ ^b^	1.19^b^	2.6^l^	1.1 × 10^−4^ ^b^	2.5 × 10^−5^ ^b^	2.0 × 10^−7^ ^c^	6.0 × 10^−6^ ^j^	7.8 × 10^−6^ ^b^	4.0 × 10^−6^ ^g^	0^d^
	1^d^	0.01^c^	0.01^c^	1.62^b^		3.6 × 10^−5^ ^b^	1.0 × 10^−5^ ^d^	1.3 × 10^−5^ ^c^	5.7 × 10^−6^ ^k^	4.9 × 10^−4^ ^c^		40 × 10^−6^ ^g^
	1^e^	0.05^d^	0.08^c^	0.8^c^		2.5 × 10^−3^ ^b^	5.1 × 10^−4^ ^e^	3.8 × 10^−6^ ^d^	1.1 × 10^−6^ ^k^	1.0 × 10^−5^ ^c^		
	1^f^	0.02^e^	0.34^e^	1.9^c^		5.0 × 10^−5^ ^c^	4.0 × 10^−4^ ^f^	4 × 10^−6^ ^d^		5.7 × 10^−4^ ^d^		
	1^g^	0.14^f^		0.8^d^		1.0 × 10^−5^ ^d^	5.0 × 10^−4^ ^g^	1 × 10^−6^ ^d^		2.1 × 10^−4^ ^e^		
	1^h^	0.096^g^		1.20^d^		7.8 × 10^−6^ ^d^	2.1 × 10^−4^ ^h^			1.6 × 10^−4^ ^f^		
	1^i^	0.1^k^		—		2.4 × 10^−5^ ^e^	2.3 × 10^−4^ ^i^			3.0 × 10^−5^ ^g^		

Average	1	0.11	0.11	1.25	2.6	3.9 × 10^−4^	2.7 × 10^−4^	4.4 × 10^−6^	4.3 × 10^−6^	2.1 × 10^−4^	4.0 × 10^−6^	2.0 × 10^−6^

^a^Estradiol equivalency factor; ^b^Rutishauser et al., 2004 [17]; ^c^Campbell et al., 2006 [14]; ^d^Murk et al., 2002 [20]; ^e^Sun et al., 2008 [21]; ^f^Purdom et al., 1994 [22]; ^g^Legler et al., 2002 [23]; ^h^Song et al., 2006 [24]; ^i^Viganò et al., 2008 [25]; ^j^Duft et al., 2003 [26]; ^k^Brix et al., 2010 [18]; ^l^Pojana et al., 2004 [27].

**Table 4:** Dissolved phase concentrations (ng·L^−1^) for each target compound in the three WWTPS under study.

	Jul '10	Sep '10	Nov '10	Jan '11	March '11	May '11	Jul '11	Frequency (%)	Average
WWTP 1									
NP^a^	88.5 ± 0.7	18.1 ± 1.1	71.0 ± 5.0	209.1 ± 15.3	77.0 ± 0.9	89.4 ± 9.7	110.0 ± 9.0	100	94.7
OP^a^	12.6 ± 0.7	BQL^c^	9.7 ± 0.7	95.2 ± 9.2	17.8 ± 1.1	11.7 ± 0.9	23.0 ± 0.9	85.7	28.3
NPEOs^a^	729.4 ± 0.7	395.3 ± 22.7	958.9 ± 17.3	464.3 ± 18.7	313.0 ± 12.9	303.7 ± 9.3	572.0 ± 22.3	100	533.8
OPEOs^a^	101.6 ± 0.7	94.1 ± 4.5	569.0 ± 45.9	350.4 ± 22.9	66.9 ± 5.9	84.2 ± 5.3	93.9 ± 7.3	100	194.3
BPA^a^	12.6 ± 0.7	BQL^c^	BQL^c^	9.6 ± 0.9	BDL^b^	BQL^c^	25.1 ± 3.2	42.9	15.8
E2^a^	21.3 ± 0.7	18.2 ± 0.3	39.2 ± 2.9	65.4 ± 3.1	BQL^c^	8.3 ± 0.5	14.7 ± 1.3	85.7	27.9
E1^a^	4.5 ± 0.7	3.9 ± 0.1	9.7 ± 0.9	BDL^b^	8.2 ± 0.5	BQL^c^	BDL^b^	57.1	6.6
E3^a^	16.4 ± 0.7	31.9 ± 2.1	42.1 ± 2.0	18.7 ± 0.9	19.2 ± 1.9	7.7 ± 0.3	12.8 ± 0.9	100	21.3
EE^a^	BQL^c^	2.9 ± 0.1	BQL^c^	BQL^c^	BQL^c^	BDL^b^	BQL^c^	14.3	2.9
DES^a^	BQL^c^	BQL^c^	BQL^c^	BQL^c^	BQL^c^	BQL^c^	BQL^c^	0.0	0.0

WWTP 2									
NP^a^	31.4 ± 3.1	49.3 ± 3.3	12.7 ± 0.3	95.8 ± 7.5	17.5 ± 1.1	15.7 ± 3.9	56.6 ± 2.3	100	39.9
OP^a^	BQL^c^	12.9 ± 0.7	3.1 ± 0.1	14.9 ± 0.8	11.4 ± 0.4	18.5 ± 1.1	BDL^b^	71.4	14.4
NPEOs^a^	698.0 ± 53.5	230.1 ± 13.3	534.4 ± 11.9	302.5 ± 19.6	94.0 ± 4.1	95.8 ± 6.9	178.6 ± 12.9	100	304.8
OPEOs^a^	187.1 ± 11.7	94.4 ± 7.9	195.8 ± 12.7	230.9 ± 21.5	56.6 ± 3.6	95.4 ± 8.1	53.2 ± 4.6	100	130.5
BPA^a^	18.1 ± 1.7	4.9 ± 0.2	7.4 ± 0.8	BQL^c^	BQL^c^	39.5 ± 3.1	8.0 ± 0.8	71.4	15.6
E2^a^	9.1 ± 0.1	14.8 ± 0.7	19.5 ± 1.1	26.9 ± 1.5	14.5 ± 1.5	13.5 ± 1.3	7.1 ± 0.6	100	15.1
E1^a^	1.9 ± 0.2	BQL^c^	4.9 ± 0.1	BQL^c^	5.4 ± 0.4	BQL^c^	BQL^c^	42.9	4.1
E3^a^	16.1 ± 1.3	8.3 ± 0.1	7.2 ± 0.3	18.5 ± 2.1	8.9 ± 0.2	18.0 ± 1.5	BDL^b^	85.7	12.2
EE^a^	BQL^c^	BQL^c^	14.3 ± 0.6	BQL^c^	BDL^b^	BQL^c^	BQL^c^	14.3	14.3
DES^a^	BQL^c^	BQL^c^	BQL^c^	BQL^c^	BQL^c^	BQL^c^	BQL^c^	0.0	0.0

^a^Mean and standard deviation of three determinations; ^b^concentration below the limit of detection; ^c^concentration below the limit of quantification.

**Table 5:** Sludge and particulate matter (solid fractions) concentrations (ng·g^−1^) for each target compound in both WWTPS under study.

Sludge samples	Jul '10	Sep '10	Nov '10	Jan '11	March '11	May '11	Jul '11	Frequency (%)	Average
WWTP 1									
NP^a^	216.1 ± 9.1	479.1 ± 22.7	52.7 ± 2.1	211.3 ± 5.2	324.5 ± 6.7	175.9 ± 12.3	22.1 ± 0.2	100	211.7
OP^a^	49.3 ± 1.4	22.2 ± 1.2	39.1 ± 3.0	20.7 ± 2.0	8.7 ± 0.6	14.0 ± 2.3	22.3 ± 1.0	100	25.2
NPEOs^a^	461.1 ± 15.7	890.1 ± 35.1	125.4 ± 11.1	471.5 ± 8.1	92.1 ± 6.7	92.4 ± 3.3	451.4 ± 10.1	100	369.1
OPEOs^a^	74.5 ± 6.0	55.9 ± 1.7	21.9 ± 1.2	190.1 ± 9.5	25.1 ± 1.5	129.3 ± 9.1	44.0 ± 1.3	100	77.3
BPA^a^	0.9 ± 0.1	BQL^c^	7.7 ± 0.7	BQL^c^	BQL^c^	2.5 ± 0.2	7.9 ± 0.5	57.1	4.8
E2^a^	10.1 ± 1.0	5.1 ± 0.3	14.1 ± 0.9	BQL^c^	17.9 ± 1.4	20.6 ± 1.6	39.1 ± 1.1	85.7	17.8
E1^a^	BQL^c^	BQL^c^	7.5 ± 0.3	BQL^c^	1.7 ± 0.1	BQL^c^	3.2 ± 0.3	42.9	4.1
E3^a^	BQL^c^	BQL^c^	BQL^c^	BQL^c^	BQL^c^	BQL^c^	0.3 ± 0.0	14.3	0.3
EE^a^	27.1 ± 2.1	21.1 ± 2.0	22.0 ± 1.7	5.9 ± 0.4	38.9 ± 2.1	9.3 ± 0.5	42.0 ± 2.7	100	23.8
DES^a^	BQL^c^	BQL^c^	3.0 ± 0.4	BQL^c^	0.9 ± 0.1	11.5 ± 0.7	BQL^c^	42.9	5.1

WWTP 2									
NP^a^	91.0 ± 5.0	101.2 ± 7.9	370.2 ± 26.3	250.1 ± 9.3	27.7 ± 2.1	63.0 ± 1.1	32.0 ± 1.8	100	133.6
OP^a^	60.1 ± 2.5	38.1 ± 3.1	35.1 ± 2.2	50.9 ± 1.7	12.5 ± 0.2	16.7 ± 1.3	71.2 ± 0.9	100	40.7
NPEOs^a^	240.9 ± 19.6	500.4 ± 32.3	612.1 ± 19.1	96.1 ± 1.1	218.3 ± 15.5	683.4 ± 4.5	403.9 ± 27.1	100	393.6
OPEOs^a^	311.8 ± 6.3	43.2 ± 2.1	104.0 ± 7.1	44.8 ± 2.3	47.5 ± 2.5	191.0 ± 7.0	161.6 ± 3.3	100	129.1
BPA^a^	BQL^c^	BQL^c^	15.9 ± 0.9	2.7 ± 0.2	1.9 ± 0.1	BQL^c^	7.6 ± 0.7	71.4	6.8
E2^a^	1.7 ± 0.2	7.2 ± 0.3	33.9 ± 1.0	21.1 ± 1.1	7.7 ± 0.1	73.7 ± 3.2	1.5 ± 0.1	100	21.0
E1^a^	4.3 ± 0.2	BQL^c^	5.0 ± 0.3	BQL^c^	4.5 ± 0.1	1.5 ± 0.1	BQL^c^	57.1	3.8
E3^a^	BQL^c^	BQL^c^	BQL^c^	BQL^c^	BQL^c^	BQL^c^	BQL^c^	0	0.0
EE^a^	111.1 ± 9.3	56.1 ± 2.1	121.7 ± 1.3	13.1 ± 0.4	25.8 ± 2.1	83.1 ± 3.9	55.9 ± 3.3	100	66.7
DES^a^	BQL^c^	BQL^c^	17.5 ± 0.4	BQL^c^	BQL^c^	BQL^c^	3.8 ± 0.3	28.6	10.7

Particulate matter	Jul '10	Sep '10	Nov '10	Jan '11	March '11	May '11	Jul '11	Frequency (%)	Average

WWTP 1									
NP^a^	198.6 ± 5.3	754.3 ± 39.1	105.7 ± 4.7	390.4 ± 33.3	401.6 ± 30.7	200.0 ± 15.9	90.9 ± 3.4	100	305.9
OP^a^	22.7 ± 0.9	50.0 ± 2.9	19.9 ± 1.1	120.4 ± 8.0	68.1 ± 4.3	30.5 ± 0.9	11.6 ± 1.0	100	50.1
NPEOs^a^	618.3 ± 40.2	391.8 ± 19.5	93.6 ± 6.1	290.5 ± 17.4	214.2 ± 16.1	183.5 ± 12.0	207.9 ± 10.6	100	285.7
OPEOs^a^	99.2 ± 3.9	69.6 ± 3.1	BQL^c^	78.9 ± 7.0	33.9 ± 1.9	40.7 ± 2.1	60.8 ± 5.9	85.7	63.9
BPA^a^	3.7 ± 0.2	12.3 ± 0.8	BQL^c^	BQL^c^	BQL^c^	BQL^c^	12.7 ± 0.6	42.9	9.6
E2^a^	21.5 ± 2.7	9.0 ± 0.7	51.7 ± 0.6	29.5 ± 3.3	7.1 ± 2.0	31.8 ± 1.3	50.3 ± 3.0	100	28.7
E1^a^	22.5 ± 0.9	14.0 ± 0.9	BQL^c^	5.1 ± 0.4	BQL^c^	11.0 ± 0.8	BQL^c^	57.1	13.2
E3^a^	BQL^c^	BQL^c^	BQL^c^	3.1 ± 0.1	BQL^c^	BQL^c^	BQL^c^	14.3	3.1
EE^a^	14.0 ± 0.7	BQL^c^	7.7 ± 0.4	BQL^c^	105.1 ± 7.8	12.9 ± 1.0	19.1 ± 0.5	71.4	31.8
DES^a^	14.3 ± 1.0	BQL^c^	6.3 ± 0.3	BQL^c^	10.4 ± 0.4	BQL^c^	BQL^c^	42.9	10.3

WWTP 2									
NP^a^	129.2 ± 7.9	212.4 ± 3.9	209 ± 9.6	301.8 ± 19.1	63.6 ± 2.9	331.9 ± 20.1	99.5 ± 1.8	100	192.5
OP^a^	34.8 ± 0.7	41.6 ± 2.9	49.0 ± 3.3	69.7 ± 3.8	5.0 ± 0.2	22.9 ± 1.4	17.7 ± 0.9	100	34.4
NPEOs^a^	89.5 ± 5.3	350.1 ± 3.1	189.6 ± 10.6	78.0 ± 2.0	189.2 ± 4.9	1003 ± 61.9	106.8 ± 14.7	100	286.6
OPEOs^a^	129.6 ± 11.8	51.0 ± 2.1	128.4 ± 5.1	77.1 ± 0.9	31.7 ± 0.9	59.7 ± 5.1	184.6 ± 3.9	100	100.4
BPA^a^	21.5 ± 1.5	BQL^c^	BQL^c^	BQL^c^	3.9 ± 0.6	BQL^c^	BQL^c^	28.6	12.7
E2^a^	33.0 ± 2.0	BQL^c^	33.7 ± 2.9	22.7 ± 0.7	14.6 ± 0.4	44.8 ± 0.7	25.1 ± 3.3	85.7	29.0
E1^a^	13.5 ± 0.3	5.9 ± 0.3	21.3 ± 2.0	BQL^c^	BQL^c^	32.7 ± 0.8	5.5 ± 0.2	57.1	15.8
E3^a^	BQL^c^	BQL^c^	BQL^c^	BQL^c^	BQL^c^	BQL^c^	BQL^c^	0	0.0
EE^a^	33.9 ± 0.9	12.0 ± 0.1	75.9 ± 2.7	29.0 ± 3.1	91.8 ± 2.9	29.4 ± 0.5	BQL^c^	85.7	45.3
DES^a^	4.1 ± 0.3	BQL^c^	23.7 ± 2.0	BQL^c^	BQL^c^	6.9 ± 0.1	BQL^c^	42.9	11.6

^a^Mean and standard deviation of three determinations; ^b^concentration below the limit of detection; ^c^concentration below the limit of quantification.

## Abbreviations

AP_*n*_EOs: Alkylphenolic polyethoxylated surfactants
BBAs: Biological based assays
BMR: Biomembrane reactor
BPA: Bisphenol-A
CAS: Conventional activated sludge
DES: Diethylstilbestrol
E1: Estrone
E2: 17*β*-estradiol
E3: Estriol
EC_50_: Half maximal effective concentration
EE: 17*α*-Ethinylestradiol
EDC: Endocrine-disrupting compound
e-EDC: Oestrogenic endocrine-disrupting compound
EEF: Oestradiol equivalency factor
EEQ: Oestradiol equivalent concentration
ER: Oestrogen receptor
ESI: Electrospray ionization
hER*α*: Human oestrogen receptor
HPLC: High performance liquid chromatography
*K*_ow_: Octanol/Water partition coefficient
LC: Liquid chromatography
LOD: Limit of detection
MAE: Microwave-assisted extraction
MeOH: Methanol
MRM: Multiple reaction monitoring
MS: Mass spectrometry
NP: Nonylphenol
NPEOs: Nonylphenolic polyethoxylated surfactants
OP: Octylphenol
OPEOs: Octylphenolic polyethoxylated surfactants
PTFE: Polytetrafluoroethylene
RP: Reversed phase
SPE: Solid phase extraction
UAE: Ultrasonic assisted extraction
UHPLC: Ultra-high performance liquid chromatography
WWTPs: Wastewater treatment plants
YES: Yeast oestrogen screen.

## References

[1] Baker VA (2001). Endocrine disrupters—testing strategies to assess human hazard. *Toxicology in Vitro*.

[2] Fisher JS (2004). Are all EDC effects mediated via steroid hormone receptors?. *Toxicology*.

[3] Welshons WV, Thayer KA, Judy BM, Taylor JA, Curran EM, vom Saal FS (2003). Large effects from small exposures. I. Mechanisms for endocrine-disrupting chemicals with estrogenic activity. *Environmental Health Perspectives*.

[4] Sonnenschein C, Soto AM (1998). An updated review of environmental estrogen and androgen mimics and antagonists. *Journal of Steroid Biochemistry and Molecular Biology*.

[5] Gleason TR, Nacci DE (2001). Risks of endocrine-disrupting compounds to wildlife: extrapolating from effects on individuals to population response. *Human and Ecological Risk Assessment*.

[6] Yang M, Park MS, Lee HS (2006). Endocrine disrupting chemicals: human exposure and health risks. *Journal of Environmental Science and Health C*.

[7] Toppari J, Larsen JC, Christiansen P (1993). Steroid hormone receptors: interaction with deoxyribonucleic acid and transcription factors. *Endocrine Reviews*.

[8] Fernández MF, Olmos B, Granada A (2007). Human exposure to endocrine-disrupting chemicals and prenatal risk factors for cryptorchidism and hypospadias: a nested case-control study. *Environmental Health Perspectives*.

[9] Sharpe RM (1993). Declining sperm count in men—is there an endocrine cause?. *Journal of Endocrinology*.

[10] Kavlock RJ, Ankley GT (1996). A perspective on the risk assessment process for endocrine-disruptive effects on wildlife and human health. *Risk Analysis*.

[11] Norris DO, Carr JA (2006). *Endrocrine Disrupters—Biological Bases For Health Effects in Wildlife and Humans*.

[12] Vos JG, Dybing E, Greim HA (2000). Health effects of endocrine-disrupting chemicals on wildlife, with special reference to the European situation. *Critical Reviews in Toxicology*.

[13] Clotfelter ED, Bell AM, Levering KR (2004). The role of animal behaviour in the study of endocrine-disrupting chemicals. *Animal Behaviour*.

[14] Campbell CG, Borglin SE, Green FB, Grayson A, Wozei E, Stringfellow WT (2006). Biologically directed environmental monitoring, fate, and transport of estrogenic endocrine disrupting compounds in water: a review. *Chemosphere*.

[15] Körner W, Bolz U, Süßmuth W (2000). Input/output balance of estrogenic active compounds in a major municipal sewage plant in Germany. *Chemosphere*.

[16] Wagner M, Oehlmann J (2000). Endocrine disruptors in bottled mineral water: total estrogenic burden and migration from plastic bottles. *Chemosphere*.

[17] Rutishauser BV, Pesonen M, Escher BI (2004). Comparative analysis of estrogenic activity in sewage treatment plant effluents involving three in vitro assays and chemical analysis of steroids. *Environmental Toxicology and Chemistry*.

[18] Brix R, Postigo C, González S (2010). Analysis and occurrence of alkylphenolic compounds and estrogens in a European river basin and an evaluation of their importance as priority pollutants. *Analytical and Bioanalytical Chemistry*.

[19] Vega-Morales T, Sosa-Ferrera Z, Santana-Rodríguez JJ (2012). Development and optimisation of an on-line solid phase extraction coupled to ultra-high-performance liquid chromatography-tandem mass spectrometry methodology for the simultaneous determination of endocrine disrupting compounds in wastewater samples. *Journal of Chromatography A*.

[20] Murk AJ, Legler J, van Lipzig MMH (2002). Detection of estrogenic potency in wastewater and surface water with three in vitro bioassays. *Environmental Toxicology and Chemistry*.

[21] Sun Q, Deng S, Huang J, Shen G, Yu G (2008). Contributors to estrogenic activity in wastewater from a large wastewater treatment plant in Beijing, China. *Environmental Toxicology and Pharmacology*.

[22] Purdom CE, Hardiman PA, Bye VJ, Eno NC, Tyler CR, Sumpter JP (1994). Estrogenic effects of effluents from sewage treatment works. *Chemistry and Ecology*.

[23] Legler J, Zeinstra LM, Schuitemaker F (2002). Comparison of in vivo and in vitro reporter gene assays for short-term screening of estrogenic activity. *Environmental Science and Technology*.

[24] Song M, Xu Y, Jiang Q (2006). Measurement of estrogenic activity in sediments from Haihe and Dagu River, China. *Environment International*.

[25] Viganò L, Benfenati E, Cauwenberge AV (2008). Estrogenicity profile and estrogenic compounds determined in river sediments by chemical analysis, ELISA and yeast assays. *Chemosphere*.

[26] Duft M, Schulte-Oehlmann U, Weltje L, Tillmann M, Oehlmann J (2003). Stimulated embryo production as a parameter of estrogenic exposure via sediments in the freshwater mudsnail Potamopyrgus antipodarum. *Aquatic Toxicology*.

[27] Pojana G, Bonfà A, Busetti F, Collarin A, Marcomini A (2004). Estrogenic potential of the Venice, Italy, lagoon waters. *Environmental Toxicology and Chemistry*.

[28] Vega-Morales T, Sosa-Ferrera Z, Santana-Rodríguez JJ (2011). Determination of various estradiol mimicking-compounds in sewage sludge by the combination of microwave-assisted extraction and LC-MS/MS. *Talanta*.

[29] Vega-Morales T, Sosa-Ferrera Z, Santana-Rodríguez JJ (2010). Determination of alkylphenol polyethoxylates, bisphenol-A, 17*α*-ethynylestradiol and 17*β*-estradiol and its metabolites in sewage samples by SPE and LC/MS/MS. *Journal of Hazardous Materials*.

[30] Céspedes R, Petrovic M, Raldúa D (2004). Integrated procedure for determination of endocrine-disrupting activity in surface waters and sediments by use of the biological technique recombinant yeast assay and chemical analysis by LC-ESI-MS. *Analytical and Bioanalytical Chemistry*.

[31] Aerni H-R, Kobler B, Rutishauser BV (2004). Combined biological and chemical assessment of estrogenic activities in wastewater treatment plant effluents. *Analytical and Bioanalytical Chemistry*.

[32] Petrović M, Barceló D (2000). The stability of non-ionic surfactants and linear alkyl sulfonates in a water matrix and on solid-phase extraction cartridges. *Fresenius’ Journal of Analytical Chemistry*.

[33] Céspedes R, Lacorte S, Ginebreda A, Barceló D (2008). Occurrence and fate of alkylphenols and alkylphenol ethoxylates in sewage treatment plants and impact on receiving waters along the Ter River (Catalonia, NE Spain). *Environmental Pollution*.

[34] Gutendorf B, Westendorf J (2001). Comparison of an array of in vitro assays for the assessment of the estrogenic potential of natural and synthetic estrogens. *Toxicology*.

[35] Legler J, Dennekamp M, Vethaak AD (2002). Detection of estrogenic activity in sediment-associated compounds using in vitro reporter gene assays. *Science of the Total Environment*.

[36] Pojana G, Gomiero A, Jonkers N, Marcomini A (2007). Natural and synthetic endocrine disrupting compounds (EDCs) in water, sediment and biota of a coastal lagoon. *Environment International*.

[37] Ying G-G, Williams B, Kookana R (2002). Environmental fate of alkylphenols and alkylphenol ethoxylates—a review. *Environment International*.

[38] Petrovic M, Diaz A, Ventura F, Barceló D (2001). Simultaneous determination of halogenated derivatives of alkylphenol ethoxylates and their metabolites in sludges, river sediments, and surface, drinking, and wastewaters by liquid chromatography-mass spectrometry. *Analytical Chemistry*.

[39] Langford KH, Lester JN (2002). *Endocrine Disrupters in Wastewater and Sludge Treatment Processes*.

[40] Andreu V, Ferrer E, Rubio JL, Font G, Picó Y (2007). Quantitative determination of octylphenol, nonylphenol, alkylphenol ethoxylates and alcohol ethoxylates by pressurized liquid extraction and liquid chromatography-mass spectrometry in soils treated with sewage sludges. *Science of the Total Environment*.

[41] Hansen P-D, Dizer H, Hock B (1998). Vitellogenin—a biomarker for endocrine disruptors. *Trends in Analytical Chemistry*.

[42] Ying G-G, Kookana RS (2005). Sorption and degradation of estrogen-like-endocrine disrupting chemicals in soil. *Environmental Toxicology and Chemistry*.

[43] Ying G-G, Kookana RS (2003). Degradation of five selected endocrine-disrupting chemicals in seawater and marine sediment. *Environmental Science and Technology*.

